# Management of surgical diseases of Primary Hyperparathyroidism: indications of the United Italian Society of Endocrine Surgery (SIUEC)

**DOI:** 10.1007/s13304-024-01796-5

**Published:** 2024-04-15

**Authors:** Paolo Del Rio, Marco Boniardi, Loredana De Pasquale, Giovanni Docimo, Maurizio Iacobone, Gabriele Materazzi, Fabio Medas, Michele Minuto, Barbara Mullineris, Andrea Polistena, Marco Raffaelli, Pietro Giorgio Calò

**Affiliations:** 1 Unit of General Surgery, Department of Medicine and Surgery, University of Parma, Parma, Italy; 2 Endocrine Surgery Unit, Department of General Oncology and Mini-Invasive Surgery, ASST Grande Ospedale Metropolitano Niguarda, 20162 Milan, Italy; 3 Thyroid and Parathyroid Unit, Otolaryngology Unit, Department of Health Sciences, ASST Santi Paolo E Carlo, Università Degli Studi Di Milano, Milan, Italy; 4 Division of Thyroid Surgery, University of Campania “L. Vanvitelli”, Naples, Italy; 5 Endocrine Surgery Unit, Department of Surgery, Oncology and Gastroenterology, Padova University Hospital, Padua, Italy; 6 Endocrine Surgery Unit, University Hospital of Pisa, Pisa, Italy; 7 Department of Surgical Sciences, University of Cagliari, Cagliari, Italy; 8 Department of Surgical Sciences and Integrated Diagnostics, University of Genoa, IRCCS Ospedale Policlinico San Martino, Genoa, Italy; 9 Unit of General Surgery, Emergency and New Technologies, Modena Hospital, 41126 Modena, Italy; 10 Department of Surgery, University of Rome Sapienza, Policlinico Umberto I, Rome, Italy; 11 Centro Dipartimentale Di Chirurgia Endocrina E Dell’Obesità, U.O.C. Chirurgia Endocrina E Metabolica, Fondazione Policlinico Universitario Agostino Gemelli IRCCS, Rome, Italy; 12 Centro Di Ricerca in Chirurgia Delle Ghiandole Endocrine E Dell’Obesità, Università Cattolica del Sacro Cuore, Rome, Italy

**Keywords:** Italian Society of Endocrine Surgery, Recommendations, Surgical diseases of parathyroid glands, Parathyroidectomy

## Abstract

A task force of the United Italian society of Endocrine Surgery (SIUEC) was commissioned to review the position statement on diagnostic, therapeutic and health‑care management protocol in parathyroid surgery published in 2014, at the light of new technologies, recent oncological concepts, and tailored approaches. The objective of this publication was to support surgeons with modern rational protocols of treatment that can be shared by health-care professionals, taking into account important clinical, healthcare and therapeutic aspects, as well as potential sequelae and complications. The task force consists of 12 members of the SIUEC highly trained and experienced in thyroid and parathyroid surgery. The main topics concern diagnostic test and localization studies, mode of admission and waiting time, therapeutic pathway (patient preparation for surgery, surgical treatment, postoperative management, management of major complications), hospital discharge and patient information, outpatient care and follow-up, outpatient initial management of patients with pHPT.

## Introduction

The United Italian Society of Endocrine Surgery (SIUEC) was established in Bari (Italy) in 2017 from the union of two pre‑existing societies (the Italian Society of Endocrine Surgery, SIEC and the Italian Association of Endocrine Surgery Units, U.E.C. CLUB). Following the long history and activities of the two founder societies, SIUEC promoted in the last 6‑year several conferences, congresses, scientific publications and professional training activities. There is a specific need to define and promote among the entire endocrine surgical community in Italy updated recommendations for the diagnosis and management of surgical parathyroid diseases, according to international protocols as recently published for thyroid surgery [1]. At the light of new technologies, new clinical, diagnostic concepts and recent pathological classification and based on the previous experience of the II Consensus Conference of the Italian Association of Endocrine Surgery Units (U.E.C. CLUB) on the Diagnostic, therapeutic and healthcare management protocols in parathyroid published in 2014 [2], an updated revision of the previous protocol was drafted by a SIUEC experts’ commission to provide clear clinical indications for endocrine surgeons in Italy related to primary Hyperparathyroidism (pHPT). The objective of this publication is to support surgeons with modern rational protocols of treatment that can be shared by health‑care professionals, taking into account important clinical, healthcare and therapeutic aspects, as well as potential sequelae and complications.

This publication aims to support the endocrine surgeons in daily practice. However, it is not within the scope of the authors nor of the SIUEC to influence in any way the physician–patient relationship, which is based on trust and clinical judgment in each individual case.

The following topics were examined:

- Diagnostic test and localization studies;
- Mode of admission and waiting time;
- Therapeutic pathway:

- patient preparation for surgery.
- surgical treatment.
- postoperative management.
- Management of major complications.

- Hospital discharge and patient information;
- Outpatient initial management of patients with pHPT.
- Outpatient care and follow-up.

### Diagnostic test and localization studies

Diagnostic tests should be worthwhile and not only aimed at the nosographic definition of pHPT (laboratory assessment), but also at localizing the affected parathyroid glands (instrumental investigations) and at defining a therapeutic indication.

#### Laboratory assessment

- First tier assessments

- serum calcium (total or ionized calcium). Measurement of ionized calcium levels may be useful in case of altered plasma albumin levels [3];
- serum phosphorus;
- serum creatinine;
- intact PTH (1–84) (second- or third generation assay);
- 25-OH-Vitamin D [4].

- Second tier assessments

- 24 h calciuria and phosphaturia;
- Creatinine clearance (calculate the ratio of calcium clearance and creatinine clearance, for differential diagnosis with Familial Hypocalciuric Hypercalcemia).

In selected cases (patients aged\30 years at diagnosis, family history of hypercalcemia, neuroendocrine tumours), assessing the presence of a familial syndrome (Multiple Endocrine Neoplasia 1––MEN1, MEN2A, Hyperparathyroidism.

jaw Tumour Syndrome, Familial Hyperparathyroidism), also by employing DNA sequencing for mutations of *RET, CASR, MEN1, CDC73* may be useful [4–6].

It is of utmost importance to underline that localization studies have no role in the diagnosis of hyperparathyroidism; nevertheless, a correct preoperative localization of pathologic parathyroids is essential to plan a correct surgical strategy (minimally invasive vs. traditional surgery).

Nowadays, minimally invasive or “focused” parathyroidectomy (no matter which technique is used) is favoured over the traditional bilateral parathyroid exploration, because of its limited surgical dissection, decreased operative time and decreased rate of complications. A minimally invasive parathyroidectomy can be indicated when: 1) two different localization studies (US and [99mTc]Tc-MIBI scintigraphy or Choline-PET) show concordant positive results or, 2) a single localization study shows a positive finding and intraoperative PTH is available during surgery. In all other cases, bilateral parathyroid exploration still remains the procedure of choice, to guarantee the highest cure rate [7, 8].

Studies available to identify hyperfunctioning parathyroids (it is important to remind that normal parathyroids are basically invisible to any imaging study) include: neck US (a morphological study) and radionuclide imaging (that includes various functional studies, such as [99mTc]Tc-MIBI scintigraphy and Choline-PET).

Both morphological and functional studies have advantages and limitations: US (especially when performed by the operating surgeon) allows a detailed anatomical localization of the parathyroid(s) and the best evaluation of the thyroid gland, and is considered essential in the planning of the surgery. Nuclear medicine techniques have the advantage of allowing the identification of ectopic glands either superiorly or inferiorly located. Both US and functional studies may have false positive and false negative results, although the techniques are often complementary. It is important to underline that, in patients with pHPT, if imaging studies are negative or discordant, the risk of multiglandular parathyroid disease is significantly increased [9].

Neck US allows a correct identification of the hyperfunctioning parathyroid(s) in 74–90% of patients, with a sensitivity of 87% and a specificity of 88%, in the hands of the endocrine surgeon [10]. In general, it allows also a characterization of the thyroid gland that is useful in the diagnosis of thyroid nodules and the indication of FNA, as well as in the identification and characterization of neck lymph nodes. In highly selected cases (e.g., in cases of persistent/recurrent hyperparathyroidism) and in experience hands, US might guide an FNA examination on the suspicious parathyroid lesion.

## Nuclear medicine imaging:

*[99mTc]Tc-MIBI scintigraphy*, especially when associated with computed tomography (SPECT-CT) is the most widely used functional imaging to localize hyperfunctioning parathyroids. The tracers accumulate in hyperfunctioning parathyroids because of their increased number of mitochondria, considered to be related to the number of oxyphilic parathyroid cells. It also accumulates in the thyroid cells, but its washout from the thyroid gland is generally (although not always) faster than from the parathyroid glands. This technique showed a detection rate for hyperfunctioning parathyroids of 88%, in a meta-analysis including 1236 patients with primary hyperparathyroidism [11].

The main limit of SPECT-CT is multiglandular disease, its sensitivity ranging from 27 to 61% against 93 and 97% for single-gland disease [12, 13].

There are currently two modalities of acquisition for SPECT-CT: dual phase parathyroid scan (that relies upon the different washout of the tracer in the thyroid and the parathyroids, a scan that needs 90–150’ to be completed) and dual-tracer parathyroid scan (possibly more helpful when thyroid nodules are present, and faster than the former one). Both protocols have excellent results in terms of identification of hyperfunctioning parathyroids, but dual tracer scans add additional irradiation to the thyroid.

Similarly to US, parathyroid scintiscans should always be visualized by the operating surgeon prior to surgery, to plan the correct surgical strategy and interpret also “negative” images [14].

***Positron Emission Tomography (PET).*** This technique adds a higher spatial resolution in the detection of parathyroids that are not visualized (or are doubtful) by SPECT-CT. The most commonly used tracer is [18F]-Choline (18F-Fluorocholine PET/CT), whose uptake is probably related to PTH secretion in patients with PHPT. Images acquisition should start one hour after tracer injection (possibly preceded by a first acquisition 5 min after the injection to identify also lesion with high uptake in early phase only). A recent metanalysis reported excellent results in terms of sensitivity (95%), positive predictive value (97%) and detection rate (91%) of radiolabelled choline-PET [15].

From a surgical point of view, it is worth noticing that a study from Hocevar et al. reported excellent results obtained by 18F-Fluorocholine PET/CT prior to surgery: from their data, patients with the identification of a single gland with this scan can safely undergo a focused parathyroidectomy without the use of intraoperative PTH, since the localization was accurate in 97% of patients [16].

18F-Fluorocholine PET/CT currently represents a widespread and promising technique for localizing hyperfunctioning parathyroids. When US or nuclear medicine techniques fail to identify with good precision a pathologic parathyroid in patients with PHPT, four-dimensional CT (4D-CT) or thin-cut CT scan can be used. These imaging can obtain better results in cases of small parathyroid adenomas or ambiguous location.

### Mode of admission and waiting time

#### Priority for hospital admission

- Very high: patients with severe hypercalcemia (serum calcium ≥ 14 mg/dl) require immediate in-hospital management with fluid resuscitation, followed by parathyroidectomy as soon as possible;
- High: within 1 month for patients with high suspicion of parathyroid malignancy or moderate hypercalcemia (serum calcium ≥ 12 and < 14 mg/dl);
- Intermediate: within 3 months for patients with chronic kidney disease, on transplant waiting list, or with severe osteoporosis, requiring surgical treatment or lithotripsy for renal lithiasis, or in patients affected from other malignancies waiting for adjuvant or neoadjuvant therapy; mild hypercalcemia (serum calcium ≥ 10.5 and < 12 mg/dl), especially in advanced age and during summer (risk of dehydration and progression to moderate or severe hypercalcemia);
- Low: within 6 months for benign, non-invalidating disease, in the presence of clinical conditions for surgery, e.g., age < 50 years.

#### Preoperative work up (prior to or at admission)

- Blood chemistry;
- ECG;
- Chest X-ray (when indicated, depending on patient’s age and comorbidities);
- Anesthesiological consultation;
- ENT consultation for the assessment of vocal cord mobility is strongly recommended, either through fiberoptic laryngoscopy, or, in centers with appropriate experience, through translaryngeal ultrasound [17, 18].

#### Recommendations for patients:

- Patients should follow a diet containing a moderate amount of calcium, and drink at least 2 L of water per day. A rigorous calcium-poor diet is not recommended, since it could lead to further bone demineralization and increased PTH levels. Patients with 25(OH) vitamin D deficiency (< 20 ng/ml) can start vitamin D supplementation, with short-test serum calcium testing.
- The suspension of acetyl salicylic acid (ASA) is not recommended; antiplatelet therapy (e.g., clopidogrel) should be suspended 7 days before surgery and reintroduced at least 24 h after surgery and once adequate haemostasis has been established. Traditional anticoagulant oral therapy (e.g., warfarin) should be suspended approximately 5 days before surgery and substituted with LWMH. Treatment with new anticoagulant drugs (e.g., apixaban, rivaroxaban) should be suspended 24–48 h before surgery and reintroduced as soon as possible, usually 24–48 h after surgery and once adequate haemostasis has been established; meanwhile, post-operative prophylaxis with LWMH is not recommended.

#### Admission

- On the same day of surgery, unless otherwise indicated or needed.

### Therapeutic pathway

#### Patient preparation for surgery

- *Antibiotics*: antibiotic therapy is not required, as in thyroid surgery, except for particular cases (e.g.: severe diabetes, valvular disease, immune deficiency, transplant patients) [19, 20];
- *Antithrombotic prophylaxis*: antithrombotic prophylaxis should be considered only for surgeries lasting > 45 min and in patients aged > 40 years, or in cases of comorbidities requiring LMWH (e.g., previous thromboembolic event). In this case, low-dose low molecular weight heparin (2,000–4,000 IU/day) could be administered [21].
- *Blood units*: as in thyroid surgery, predeposit of autologous blood donation or preparation of blood units is not required;
- *Position* on the operation table (joint responsibility of surgeon and anesthesiologist):

- Patient should lie in the supine position with a small wedge beneath the shoulders at the scapular level to allow mild hyperextension of the neck (not necessary during minimally invasive and remote site surgical procedures);
- Arms should be secured next to the patient’s body to avoid rare, but severe and sometimes irreversible, brachial plexus paralyses due to stretch injury;
- Elbows should be adequately padded to avoid ulnar nerve paralysis due to compression;
- Eye protection to avoid corneal ulceration and ocular trauma.

#### Informed consent

Patients should be adequately informed by the surgeon of the indications for surgery, possible alternative treatments, and the expected advantages from surgery, including improvements in long-term quality of life, general and specific complications and possible rehabilitation therapy, as well as of the clinical consequences of potential permanent postoperative injuries [22, 23].

The information provided should be clearly explained, complete and prompt. After providing the most complete information, the physician will seek the patient’s consent to perform surgery, especially taking into full consideration any expression of dissent, even on individual aspects of the procedure or its potential consequences. It is important to underline that proper informed consent may limit medicolegal claims in light of the new regulation of medical professional legal responsibility [24].

Transmission of information and informed consent should preliminarily take place at the initial outpatient visit and be renewed at admission (before surgery), especially if enough time has passed such that the initial conditions may have changed. In fact, the patient must be given the opportunity to discuss in depth with his/her physician (or other trusted person) the information received and, if desired, to obtain information on the health facility where he or she will be treated and/or on the team that will perform the surgery. It would be advisable to give the patients informative materials regarding the type of intervention, including the surgical approach (e.g., mini-invasive or robotic surgery; unilateral or bilateral exploration; use of intra-operative PTH assay) and possible postoperative complications. It is necessary that the surgeon states clearly its experience and the incidence of postoperative complications in his own operative unit.

Given the peculiarity of the therapeutic intervention (partial or total removal of parathyroid glands) and its potential consequences on the physical integrity of the subject, it is necessary that written documentation of the informed and conscious consent be retained, and that the informed consent process be documented in a specific chart note.

The following consent form should be personalized and signed off both by the patient and the physician each time:

#### Informed consent form for pHPT

*I, the undersigned, ……………………………… declare having been informed in a clear and understandable manner by Dr. ………………………….., both at initial visit and at admission, that the condition I was diagnosed with, i.e., Primary Hyperparathyroidism, requires surgical intervention. It has been explained to me that this disease is caused by one or more pathological parathyroid glands with and overproduction of parathormone and an increase of serum calcium.*

*The scope, benefits (also relative to alternative treatments), possible risks and/or foreseeable injuries have been clearly explained to me. It has been explained to me that, if ultrasound and/or scintigraphic findings will be confirmed intraoperatively, the scheduled surgery will consist of removal of the diseased gland or glands, or subtotal or total removal of parathyroid glands in the event that all glands are affected.*

*The surgical approach will consist of*

- traditional cervicotomy, ○ video-assisted parathyroidectomy, ○ trans-axillary robotic parathyroidectomy.

*The surgical approach could be varied during surgery (e.g.,: prosecution of a mini-invasive procedure with a traditional cervicotomy) in case the pathological gland could not be localised.*

*I have been informed that this procedure may involve:*

*Persistent or recurrent hyperparathyroidism if intraoperative detection of the affected gland is not possible, or in case one or more supernumerary and/or ectopic diseased parathyroid glands remain undetected.*

*Temporary or permanent injury to the laryngeal nerves that innervate the vocal cords, with sometimes severe voice alterations. In case of bilateral laryngeal nerve injury, breathing difficulties may arise that may necessitate tracheostomy, which is nearly always temporary. Voice alterations may include hoarseness of the voice, breathy, diplophonic (double-toned), high-pitch voice, as well as changes in timbre, tone, extension, intensity and fatigue in vocal use, with singing difficulties. Difficulty swallowing liquids that is usually transient may accompany these alterations.*

*Temporary or permanent injury to the explored parathyroid glands or to the gland stump (sub-total parathyroidectomy), with subsequent alterations in calcium and phosphorus blood levels requiring calcium and vitamin D supplementation, possibly for life.*

*Need to remove one or both thyroid lobes if the surgeon suspects that a parathyroid gland is located within the thyroid, if malignant disease is suspected or in case of concomitant thyroid disease necessitating surgical removal of the gland. In the latter case, lifelong thyroid hormone replacement therapy will be needed.*

*Need to remove partially or completely the thymus gland.*

*Postoperative bleeding that could require reintervention for hemostasis.*

*Wound infection.*

*The surgeon has sufficiently informed me about the incidence of these complications (also referring to his/her own experience), and has explained to me that surgery, and parathyroid surgery in particular, cannot be considered as devoid of risks even when performed with rigorous technique, since the laryngeal nerves and parathyroid glands may be temporarily or permanently injured due to causes (nerve exposure, scarring, cold- or heat-induced nerve damage, vascular damage and other unknown causes) that are independent of a correct execution of the surgical procedure.*

*I have also been told that I will have a surgical scar on my neck.*

*In any case, I am aware that if the need to rescue me from an immediate, otherwise unavoidable danger arises that could cause serious injury to myself, or if difficulties are encountered with the planned technique during surgery, the surgical team will perform all the procedures they deem necessary to prevent or reduce the harm, and to conclude the surgical procedure in the safest conditions, varying the nature of the planned procedure if necessary.*

*Now, therefore, I hereby declare that I have been asked to read carefully the content of this two-page form, which actually corresponds to what I have been extensively told. I hereby declare that I understand the meaning of what has been explained to me and that I do not need further clarifications beyond those I asked for, which I have written with my own hand below: ………………………………………………………………………………………………………………………………………..*

*Now, therefore, I consciously consent / do not consent to the proposed surgical procedure.*

*I am aware that I may withdraw this consent at any time, by telling the physicians in charge of my care.*

*Patient’s legible signature.......................................*

*Physician’s legible signature.........................................*

*P.S.: I hereby authorize / do not authorize the physicians in charge of my care to treat to the best of their knowledge and belief other conditions discovered during surgery and not previously diagnosed, but requiring non-deferrable treatment due to urgent or potentially life-threatening situations, being aware that the surgical plan originally proposed and agreed upon might have to be modified.*

*Patient’s legible signature.......................................*

*Physician’s legible signature.........................................*

*Date and time.........................*

#### Surgical indications for pHPT

- Indications for parathyroidectomy (PTx):

- symptomatic primary hyperparathyroidism;
- asymptomatic primary hyperparathyroidism with at least one of the following conditions [3, 25]:

- serum calcium levels: 1.0 mg/dL above the upper limit of normal;
- creatinine clearance < 60 cc/min;
- bone mineral density: T score < -2.5 at the 3 measurement sites (lumbar spine, femur, and 1/3 distal radius)/prior fragility fracture;
- age < 50 years.

In selected cases of asymptomatic HPT I (with difficult follow-up, when the patient appears to be willing to solve the problem even though the criteria are not met), PTx may be considered even in the absence of at least one of the aforementioned criteria, if only one parathyroid gland is suspected to be affected based on preoperative localization studies (ultrasound or scintigraphy) [5].

#### Surgical treatment of pHPT

##### Single gland disease suspected on preoperative localization studies

- Minimally invasive techniques (when imaging studies allow localization of the diseased gland, with concordance between ultrasound and scintigraphy):

- open (Open Minimally Invasive Parathyroidectomy, OMIP) and intraoperative (I.O.) measurement of PTH [26];
- radio guided (Minimally Invasive Radio-guided Parathyroidectomy, MIRP) [27];
- video assisted (Minimally Invasive Video-Assisted Parathyroidectomy, MIVAP) and I.O. measurement of PTH [28];
- endoscopic, via median or lateral approach and I.O. measurement of PTH [29, 30].

Some evidence suggests that, in case of minimally invasive ‘‘focused’’ parathyroidectomy, I.O. measurement of PTH may be omitted when there is a concordance between preoperative localization studies [5, 31].

- Traditional technique (four-gland exploration).
- Remote access parathyroidectomy (RAP) has been developed in parallel with remote access thyroid surgery. RAP includes different approaches including endoscopic and robotic trans-axillary [32], anteriorchest [32], transoral [33–35], retroauricular [32], and a combination of these approaches. All these approaches have their own advantages and disadvantages. Despite their diffusion was limited since their introduction, the available data suggest that the cure rate and the safety of such techniques are similar to those of minimally invasive and conventional approaches [36]. Nonetheless, the available data are few and their definitive role in the clinical practice is still far from being defined. In addition, they harbour the risk of new, procedure-specific complications. For these reasons, they should be limited to high-volume Institutions with experienced endoscopic and endocrine surgeons. In addition, robotic procedures, via the.
- transaxillary or the transoral approaches, are usually associated with increased costs, related to the use of the robotic platform [37]. The availability of new robotic platforms in the market, by reducing the cost of the procedure, could probably allow to give further interest for robotically assisted approaches [38].

##### Multiple gland disease suspected

Bilateral neck exploration with conventional or video-assisted technique in expert hands [39, 40] (four-gland exploration: sub-total PTx, total PTx with autotransplantation and possibly, if a Tissue Bank is available, cryopreservation).

Medical therapy with calcium-mimetic agents should be considered for those patients in whom surgical treatment for any form of hyperparathyroidism is contraindicated (due to relevant comorbidities, patient’s refusal, persistent hyperparathyroidism after failure of surgery) [5, 41].

##### Parathyroid carcinoma

Parathyroid carcinoma is a rare disease that should be suspected in the presence of substantially increased serum calcium and PTH levels. It must be treated surgically, with removal of the tumour and the surrounding structures: thyroid lobe, ipsilateral parathyroid gland, and ipsilateral central compartment lymph node dissection [42], despite recent findings have somehow questioned the utility of lymph node dissection in every case [43, 44].

##### Hyperparathyroidism associated with polyendocrine syndromes (MEN)

Primary HPT is common to both MEN1 [45], or Wermer’s syndrome (with associated tumors of the pituitary gland, endocrine pancreas and digestive tract) and MEN2A [46], or Sipple’s syndrome (with associated pheochromocytoma and medullary thyroid cancer) and the recently describe MEN4 [47].

In MEN1, hyperparathyroidism should be treated first, to remove the stimulant effect of hypercalcemia on gastrin secretion. Due to hyperplasia of all parathyroid glands, hyperparathyroidism should be preferably treated with subtotal parathyroidectomy or, as a second choice, with total parathyroidectomy with autotransplantation and cryopreservation. Regardless of the technique used, the thyreo-thymic ligament and the thymic horns must also be removed because of possible supernumerary parathyroid glands [48].

In MEN2A, adrenalectomy should precede thyroidectomy. During thyroidectomy, all parathyroid glands should be explored and, as in sporadic pHPT, removal should be limited to diseased glands (removal of enlarged glands, sub-total PTx, PTx with autotransplantation and possibly cryopreservation) [46].

CDKN1B mutations cause a MEN 1-like syndrome recently described [49] named MEN 4; the phenotypic features are still undefined due to the small number of patients reported. MEN4 includes parathyroid and anterior pituitary tumors (possibly associated with adrenal, renal, and reproductive organ tumors) [50].

In such setting, HPT 1 has a high penetrance (81%) and is often the first endocrinopathy at onset and it is described as multiglandular in most cases. Surgical resection similar to that performed in MEN 1 has been proposed, even if very few data are available and the strength of recommendation for surgical management is very low [49].

Hyperparathyroidism-jaw tumor syndrome (HPT-JT) is a rare disorder mainly characterized by the development of parathyroid tumors and ossifying fibromas of the mandible and maxilla. In this hereditary forms of primary hyperparathyroidism, a single-gland parathyroid involvement has been usually documented (86.1%), whereas multiglandular involvement occurs less commonly (13.9%). Notably, this syndrome is associated with a higher prevalence of atypical adenomas and carcinomas (up to 23% of cases). Due to the rarity of the syndrome, the appropriate surgical approach is debated; although subtotal parathyroidectomy or total parathyroidectomy with auto-transplantation has been initially suggested, targeted approaches and selective parathyroidectomy have recently been proposed to minimize surgical morbidity [51].

In case of suspicious parathyroid carcinoma, it must be treated properly as reported above.

#### Surgical adjuncts

- Intraoperative PTH
  Intraoperative PTH measurement is a very useful adjunct to all minimally invasive techniques for the treatment of HPT I, with the exception of radioguided parathyroidectomy (where, however, I.O. PTH testing could confirm successful parathyroidectomy [52]. It may be omitted if there is any concordance between preoperative localization studies [5, 35].

Although a ≥ 50% drop from baseline (blood sampling before induction) in serum PTH levels 10 min after removal of the adenoma is generally considered as adequate [53–55], this interpretative criterion of I.O. PTH is characterized by false-positive results in case of multiglandular disease [55, 56].

More stringent criteria have been proposed [57, 58] that usually require a return of PTH levels to the normal range to consider the procedure successful. The usefulness of I.O. PTH is controversial in secondary and tertiary HPT. When used, a drop in serum PTH levels by at least 75% is considered satisfactory.

Parathyroid Fluorescence Indocyanine green (ICG) fluorescence and near-infrared autofluorescence are potentially helpful to aid in the intraoperative localization of parathyroid adenomas during parathyroidectomy [59, 60].

Moreover, parathyroid fluorescence has a potential utility in intraoperative localization of mediastinal parathyroid glands or during reoperative neck surgery [61]. Immunofluorescence with ICG may be particularly useful to confirm vascularization of parathyroid remnant in subtotal parathyroidectomy for parathyroid hyperplasia [62, 63].

- Drainage
  Not required after minimally invasive surgery, optional in other cases.
- Histological examination
  On cryostat-prepared sections for tissue identification and, after processing, for definite diagnosis. If I.O. PTH measurement is performed, intraoperative histological examination is not required.

#### Postoperative management

Postoperative management should be provided by qualified medical and nursing staff who has been trained to recognize and treat possible complications, such as dyspnea, bleeding, acute hypoparathyroidism.

- Nursing care:

- checkthewoundforpossible hematoma formation;
- if present, check drains for patency and proper functioning;
- immediately notify the on-call physician in case of:

- abundant blood loss from drains,
- swollen wound,
- onset of agitation, dyspnea, feeling of tightness around neck,
- signs of hypocalcemia (paresthesias, Chvostek’s and Trousseau’s signs);

- in asymptomatic patients, measure calcium levels on the first and second postoperative day;
- measurement of intact PTH levels 24 and 48 h after surgery is optional, but advisable to confirm cure.

- Medical care:

- in case of cervical hematoma with compressive symptoms, immediately remove dressing and sutures and reopen the wound at the bedside, if required by the patient’s conditions. In the meantime, on-site staff must quickly prepare the operating room and notify the surgeon for re-exploring the wound;
- wound dressing before discharge;
- drain removal (after removing suction) on the first or second postoperative day;
- in sub-total or total parathyroidectomy, calcium and vitamin D supplementation (intravenously, if necessary) should be started early after surgery (1–2 g of calcium carbonate. and calcitriol up to 2 mcg/day) [64].
- if parathyroidectomy is combined with thyroid lobectomy and isthmectomy should be evaluated TSH reflex after 30 days.

##### Management of major complications

- Hypocalcemia.

Hypocalcemia may be more or less severe, with the onset of numbness, paresthesias, muscle cramps, tetany and, in some cases, even seizures. Besides being caused by the drop in PTH, hypocalcemia may be a consequence of the hungry bone syndrome secondary to osteodystrophy, in which the bone tends to ‘‘retrieve’’ calcium from the interstitial fluid after abrupt removal of the stimulus provided by HPT. After parathyroidectomy for pHPT, frequent measurements of serum calcium levels are required to adjust treatment with intravenous calcium gluconate.

Temporary or permanent injury to the recurrent laryngeal nerve.

- If respiratory diplegia is present at the time of tracheal extubation:

- do not perform immediate tracheostomy and keep the patient intubated for 24 h. Then remove the endotracheal tube using a fiberoptic bronchoscope to verify that at least one vocal cord has functionally recovered;
- if respiratory diplegia persists, keep the patient intubated for additional 24 h;
- if respiratory diplegia persists after 48 h, perform tracheostomy.

- In case of phonatory diplegia with an adequate airway:

- do not perform tracheostomy;
- initiate speech therapy according to the timing established by the ENT surgeon/phoniatrist.

- In case of unilateral vocal cord paralysis:

- initiate speech therapy according to the timing established by the ENT surgeon/phoniatrist, possibly after documentation (video recorded fiberoptic laryngoscopy).
- In case of severe dysphagia to liquids, administer oral thickened liquids (gels) to prevent dehydration.

In any case, clinical observations as well as proposed and/ or performed treatments must be accurately noted in the patient’s chart. Unilateral and mini-invasive parathyroid explorations have the advantage to further reduce the risk of bilateral laryngeal nerve palsy.

### Hospital discharge and patient information

- Hospital discharge

- Patients may be safely discharged on the first postoperative day after uneventful selective parathyroidectomy for pHPT, especially in performed as minimally invasive surgery;
- In case of sub-total, patients may be discharged when serum calcium levels are stable and eventually adequately compensated;
- although evidence exists that outpatient parathyroid surgery may be performed [65–67], at least a one-night hospital stay is still advisable in the present national Italian setting.

In all cases postoperative serum calcium (and possibly PTH) levels should be checked at least at the first postoperative day to confirm the surgery has been curative. In case of massive and rapid decrease of serum calcium levels, they should be normalized to avoid the risk of postoperative hypocalcemic crisis with severe cramps and tetany. Low preoperative vitamin D levels and increased bone turn-over parameters are risk factors for symptomatic hungry bone syndrome [68].

Reoperative surgery and total/subtotal parathyroidectomy are risk factors for postoperative hypoparathyroidism. Supplementation with calcium and eventually active vitamin D analogues may be used to counteract the decrease of serum calcium levels.

- Patient information

- Practical advice at discharge
  It may be useful to provide the patient with a leaflet containing practical information on self-management of recovery and contact information for use in case of need for advice.
- Hospital discharge summary
  Provide the patient with a complete clinical report, signed off by the attending physician who discharges the patient, to be given to primary care and/or referral physician containing the following essential information:

- date of admission and admit diagnosis;
- main diagnostic investigations performed, with particular emphasis on those with altered results;
- date, name and description of surgical procedure;
- discharge diagnosis.
- description of the postoperative clinical course, with accurate highlighting of any complications (haemorrhage, hypocalcaemia, dysphonia);
- medications on discharge, clearly indicating posology and method of administration;
- recommended clinical and/or diagnostic follow-up. A copy of the discharge summary should be placed in the patient’s chart.

### Outpatient initial management of patients with pHPT

- Initial visits of patients referred by their primary care physician or by a specialist.

- Patients to whom surgery is proposed should be provided with the following information:

- surgical treatment of hyperparathyroidism and concomitant thyroid surgery in case of coexisting thyroid disease; intraoperative suspicion of intrathyroid localization of parathyroid affected gland or parathyroid malignancy required en-bloc excision.
- possible alternative therapies;
- advantages that surgery may offer and possible risks associated with the surgical procedure to be performed;
- clinical consequences of potential complications and possible treatments.

- The patient should be provided with a short clinical report that includes.

- medical history, with particular reference to health conditions that may require special consideration;
- physical examination;
- diagnosis;
- proposed treatment;
- ordered diagnostic testing/investigations;
- agreement or disagreement with other consultants’ reports brought by the patient for examination;
- if surgery is proposed, the information provided should be clearly noted on the report, to obtain a preliminary consent to the recommended treatment;
- application for hospital admission, including priority for admission.

### Outpatient care and follow-up

After discharge, the early postoperative course should be checked, including the control of the surgical dressing, if needed, and the absence of complications (including dysphonia, dysphagia) and symptoms related to hypocalcemia. In such cases, vocal cord motility assessment with eventual referral to speech rehabilitative treatment (logotherapy) should be considered; serum calcium levels should be monitored to eventually adapt a calcium/vitamin D supplementation.

After successful surgery, normal dietary and/or supplemental calcium and vitamin D intake should be assured.

Patients should be informed that a further tailored follow-up should be performed by the referral physician.

The postoperative follow-up should include a measurement of calcemia at least at 3 and 6 months after surgery, to confirm the cure in case of primary HPT, and should be prolonged at least up to 5 years [69].

Prolonged, even longer follow-up should be performed in young patients, in case hereditary primary HPT, in case of multiglandular, atypical or malignant parathyroid because of the risk of late and long-term recurrences [70].

Postoperative increased PTH levels in absence of inappropriately high calcium levels should not be considered signs of persistent primary HPT but may be caused by secondary HPT that not need localization studies or surgical treatments. Preoperative very high PTH or low vitamin D levels, impaired renal function, increased bone turnover and removal of large parathyroid tumors are predictive factors of increased postoperative PTH levels [71–76].

In case of persistent postoperative inappropriately high calcium/PTH levels, persistent or recurrent HPT should be suspected and the causes should be assessed and treated by expert endocrine surgeons in high-volume centres [77].

### Practical advice after parathyroidectomy

After parathyroid surgery, serum calcium levels should be monitored frequently, according to the indications provided by the physician who will monitor the postoperative course, recovery and progresses over time (follow-up). Background therapy with calcium and/or vitamin D, if prescribed, must be taken regularly to prevent hypocalcemia, which manifests as tingling in the arms, legs and lips, or as muscle spasms, particularly in the hands (tetanic spasms). Calcium tablets/sachets may be taken. After surgery, a short recovery period is usually necessary. The patient is allowed to freely move the neck, and covering a dry wound is not necessary. After the recovery period, the patient will be able to engage in all work, family and social activities without limitations, even when parathyroidectomy is combined with thyroidectomy. Changes in voice tone are possible after surgery. The voice may either be clear or weak (early fatigability, difficulties in speaking loudly, yelling or singing). Most of these effects resolve completely within a few months. From an aesthetic standpoint, a surgical wound is considered healed after about three months. During the weeks following surgery, the individual may experience difficulty swallowing (lump in the throat sensation, firm neck skin, sensation of tightness of the chest skin during swallowing). These disturbances are generally transient and caused by deep tissue scarring. Women of childbearing potential will be able to have normal pregnancies and breastfeed. Frequent monitoring of serum calcium levels will be necessary during pregnancy, as normal calcium levels are essential to normal bone formation in the foetus. For further clarification please contact the Endocrine Surgery outpatient service on ………….. between the hours of …………. and ……………, phone ……

## Data Availability

Not available.
